# Emergence and maintenance of functional modules in signaling pathways

**DOI:** 10.1186/1471-2148-7-205

**Published:** 2007-10-31

**Authors:** Orkun S Soyer

**Affiliations:** 1 The Microsoft Research – University of Trento Centre for Computational and Systems Biology (CoSBi). Piazza Manci 17, 38100 Povo (Trento), Italy

## Abstract

**Background:**

While detection and analysis of functional modules in biological systems have received great attention in recent years, we still lack a complete understanding of how such modules emerge. One theory is that systems must encounter a varying selection (i.e. environment) in order for modularity to emerge. Here, we provide an alternative and simpler explanation using a realistic model of biological signaling pathways and simulating their evolution.

**Results:**

These evolutionary simulations start with a homogenous population of a minimal pathway containing two effectors coupled to two signals via a single receptor. This population is allowed to evolve under a constant selection pressure for mediating two separate responses. Results of these evolutionary simulations show that under such a selective pressure, mutational processes easily lead to the emergence of pathways with two separate sub-pathways (i.e. modules) each mediating a distinct response only to one of the signals. Such functional modules are maintained as long as mutations leading to new interactions among existing proteins in the pathway are rare.

**Conclusion:**

While supporting a neutralistic view for the emergence of modularity in biological systems, these findings highlight the relevant rate of different mutational processes and the distribution of functional pathways in the topology space as key factors for its maintenance.

## Background

Functional modules are observed at various levels in biology, ranging from sub cellular to the ecosystem. A general definition that holds across these different levels is that a functional module is a discrete entity whose function is separable from those of other modules [1]. One straightforward example of such a module in the cell would be a distinct pathway mediating a certain physiological response. Besides the classical biochemical characterization of such pathways, recent analyses have identified many possible modules using multiple high-throughput data sources [2,3]. Analyses of various biological connectivity data have found therein patterns that are overrepresented and might correspond to small modules [4-6] (so-called motifs). Discovered mostly from connectivity and co-expression data, it is not clear whether these "structural" modules correspond to real functional modules that are possibly conserved over evolution [7,8]. So far, it has been only possible to test the functional role of such "discovered" modules in case of few motifs [9].

While such efforts to discover and characterize distinct pathways constituting functional modules continue, we still lack a clear understanding of how modularity emerges in biological systems of multiple interacting proteins. Theoretical studies in linear systems suggest that modularity might emerge as a byproduct of selection for dynamical stability [10,11]. However, the use of a purely mathematical description of stability (i.e. ability to reach steady state) in these studies might limit extending their findings to biological pathways that are known to have non-linear dynamics. Another possibility is that modularity in complex systems is selected for, because it allows a fitness benefit under varying environments [12,13]. This is in contrast to the simpler explanation that modularity emerges neutrally as a result of evolutionary processes and does not require presence of any complex selection (as in [13]). First put forward in a "thought experiment", to explain modularity in regulatory pathways and bacterial diversification [14], the neutralistic explanation is also supported by theoretical studies with simple models of regulatory networks [15].

Here, we give a detailed treatment of the role of evolutionary processes in the emergence and maintenance of functional modules in signaling pathways. We assume that signaling pathways have evolved from a simple ancestral pathway containing few non-specific proteins, some of which acted as effectors and receptors. The fitness benefit for an organism to mediate separate (and possibly dynamically different) responses to different signals would exert a constant selective pressure on such a pathway for achieving specific signal-response relations. We propose that such a constant selective pressure would then drive pathways to evolve modular structures. To test this hypothesis, we use mathematical models of signaling pathways and evolutionary simulations. Results of these simulations show that pathways evolve readily distinct sub-pathways or modules that mediate specific signal-response relations. Further analyses highlight duplications and protein recruitment as key mutational processes facilitating modularity. On the other hand, mutations leading to new interactions among existing proteins in a pathway destroy functional modules and lead to crosstalk and complex pathways. The relevant rates of these different mutational processes that shape pathway topology, and the distribution of such topologies in the topology space emerge as the key determinants for the evolution of modularity.

## Results and discussion

**Figure 1:**
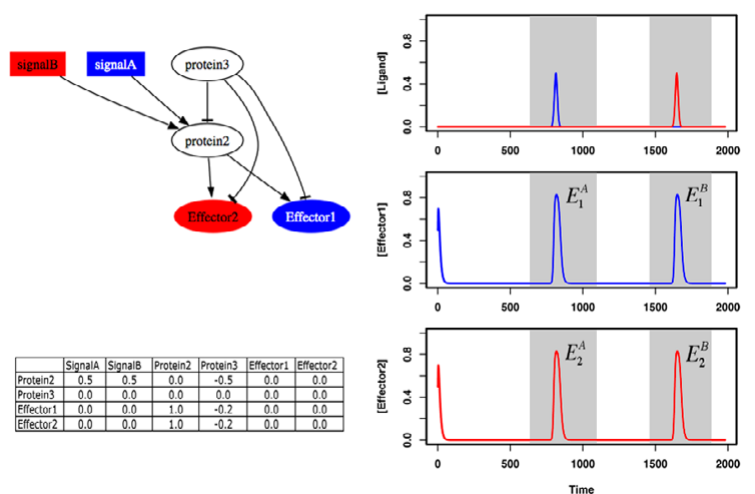
Cartoon and mathematical representation of the ancestral pathway, used in the evolutionary simulations, and its dynamical response to two ligand molecules. The latter is obtained by solving the set of differential equations describing the concentration of each protein in the pathway and is used in the calculation of pathway fitness. Gray areas indicate the time brackets when pathway response through effector 1 E1A
 MathType@MTEF@5@5@+=feaafiart1ev1aaatCvAUfKttLearuWrP9MDH5MBPbIqV92AaeXatLxBI9gBaebbnrfifHhDYfgasaacPC6xNi=xH8viVGI8Gi=hEeeu0xXdbba9frFj0xb9qqpG0dXdb9aspeI8k8fiI+fsY=rqGqVepae9pg0db9vqaiVgFr0xfr=xfr=xc9adbaqaaeGacaGaaiaabeqaaeqabiWaaaGcbaGaemyrau0aa0baaSqaaiabigdaXaqaaiabdgeabbaaaaa@2F0E@ and E1B
 MathType@MTEF@5@5@+=feaafiart1ev1aaatCvAUfKttLearuWrP9MDH5MBPbIqV92AaeXatLxBI9gBaebbnrfifHhDYfgasaacPC6xNi=xH8viVGI8Gi=hEeeu0xXdbba9frFj0xb9qqpG0dXdb9aspeI8k8fiI+fsY=rqGqVepae9pg0db9vqaiVgFr0xfr=xfr=xc9adbaqaaeGacaGaaiaabeqaaeqabiWaaaGcbaGaemyrau0aa0baaSqaaiabigdaXaqaaiabdkeacbaaaaa@2F10@) and effector 2 (E2A
 MathType@MTEF@5@5@+=feaafiart1ev1aaatCvAUfKttLearuWrP9MDH5MBPbIqV92AaeXatLxBI9gBaebbnrfifHhDYfgasaacPC6xNi=xH8viVGI8Gi=hEeeu0xXdbba9frFj0xb9qqpG0dXdb9aspeI8k8fiI+fsY=rqGqVepae9pg0db9vqaiVgFr0xfr=xfr=xc9adbaqaaeGacaGaaiaabeqaaeqabiWaaaGcbaGaemyrau0aa0baaSqaaiabikdaYaqaaiabdgeabbaaaaa@2F10@ and E2B
 MathType@MTEF@5@5@+=feaafiart1ev1aaatCvAUfKttLearuWrP9MDH5MBPbIqV92AaeXatLxBI9gBaebbnrfifHhDYfgasaacPC6xNi=xH8viVGI8Gi=hEeeu0xXdbba9frFj0xb9qqpG0dXdb9aspeI8k8fiI+fsY=rqGqVepae9pg0db9vqaiVgFr0xfr=xfr=xc9adbaqaaeGacaGaaiaabeqaaeqabiWaaaGcbaGaemyrau0aa0baaSqaaiabikdaYaqaaiabdkeacbaaaaa@2F12@) are evaluated (see *Methods*). Proteins labeled as two and three correspond to a receptor and "global deactivator" (i.e. non-specific phosphotase) respectively. Interaction coefficients are shown as a matrix, listing the actions of other proteins on a given protein row-by-row.

To test the hypothesis that modularity in signaling pathways emerges as a result of evolution towards mediating distinct responses to different signals, we use mathematical models of such pathways and simulate their evolution (see *Methods*). These simulations start with a homogenous population of an "ancestral" pathway that contains two effectors (effector one and two), one receptor and one intermediary protein. Both of these proteins are assumed to be non-specific; the receptor has equal affinity towards all ligand molecules present in the medium, and equally activates the two effectors, while the intermediary protein acts as a "global" deactivator inhibiting both the receptor and the two effectors with equal strength. Figure 1 shows this ancestral pathway and its response (the time course of active effectors) to two distinct ligand molecules (signal A and B hereafter). During the course of evolution, each generation is created from the previous one by selecting pathways randomly with replacement and allowing them to replicate with a probability proportional to fitness. Here, we use a fitness function that represents a constant selective pressure on pathways to mediate distinct responses to the different signals presented. It rewards pathways ability to respond through effector one (two) in presence of signal A (B), and not in presence of signal B (A) (see *Methods*).

**Figure 2:**
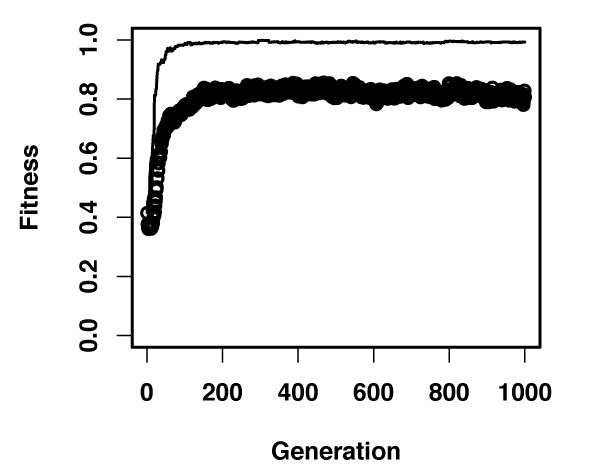
Fitness during an evolutionary simulation starting with a homogenous population containing only the ancestral pathway. Circles and the line represent the average fitness of the population and the highest fitness at each generation respectively.

**Figure 3:**
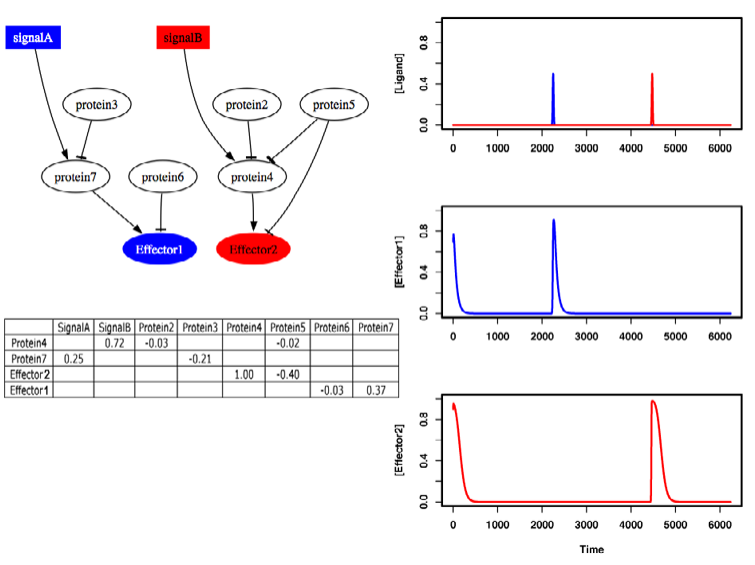
A sample pathway from the final generation of the evolutionary simulation shown in Figure 2, and its response to two ligand molecules. Note the separation of signal-response relations both at dynamic and structural levels. The mathematical description of the pathway shows only the non-zero interaction coefficients, listing the actions of other proteins on a given protein row-by-row.

Figure 2 shows the average fitness during the course of a typical evolutionary simulation. As the ancestral pathway responds in identical fashion to both signals through both effectors, the average fitness is initially low. However, evolution results quickly in high fitness values and pathways in the final population are able to respond specifically to each signal through the corresponding effectors. Figure 3 shows a sample pathway from the final population and its response. As clearly seen in the cartoon representation of this pathway, signals A and B are propagated through the pathway via receptors and over intermediary proteins to the two effectors, following two separate paths. The ancestral pathway has evolved into two separate sub-pathways or modules for processing each of the signals. In fact, such modularity is found in all pathways present in the final population. For each of these pathways there exist a path, connecting signal A (B) with effector one (two), while there is no such path to effector B (A). Additional simulations result in similar fitness curves (see Additional File 1) and final populations that contain only modular pathways. Furthermore, we find that in all these simulations modular, high fitness pathways first emerge in the population after only few generations (19 generations for the simulation shown in Figure 2). These results indicate that evolution under a constant and biologically plausible selective pressure leads readily to the emergence of functional modules in signaling pathways.

To better understand how such modularity emerges in these simulations, we analyze the evolutionary processes that shape pathway structure. Here, we consider duplication and loss of proteins, loss and formation of interactions, and adjustment of kinetic rates as such processes (see *Methods*). Formation of new interactions can result when point mutations (or accumulation thereof) on a protein lead to a new binding surface for recognizing another protein or signal, as observed *in vitro *[16,17]. Considering that there are many proteins in an organism that are not participating in a given pathway, it is much more likely that such mutations would lead to formation of a new interaction between a protein that is already participating in this pathway and one that is not (i.e. protein recruitment). This intuition leads to the assumption that formation of new interactions among existing proteins in a pathway are negligibly rare compared to new protein recruitment. Results shown in Figure 2 are obtained under such an assumption (i.e. all interaction formation events were modeled as protein recruitment. See *Methods*).

**Figure 4:**
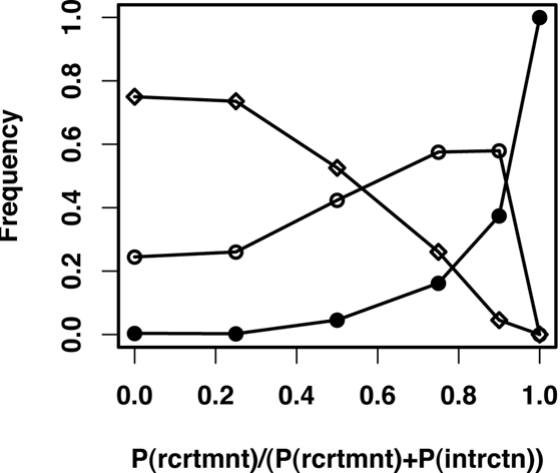
Frequency of different pathway structures in the final generation of the evolutionary simulations with increasing ratio of protein recruitment over the sum of interaction formation and protein recruitment probabilities (see *Methods*). For each probability ratio the frequencies are obtained as an average over seven different runs. We distinguish among three different structural types for pathways. Pathways where there is a path from each signal to only one effector and the other (modular, solid circles), pathways where there is a path from one of the signals to both effectors (crosstalk, open circles), pathways where there is a path from each signal to each effector (complex, open diamonds).

Relaxing this assumption, we run additional simulations with decreasing probability for protein recruitment in expense of new interactions forming among existing proteins. Figure 4 shows the frequency of different pathway types in the final populations obtained from these simulations. We find that allowing interaction formation among participating proteins in a pathway diminish the chances of modularity emerging and lead to complex pathways or crosstalk (i.e. from one of the signals there exist two paths leading to both effectors, see sample pathways shown in Additional File 2). This effect is still visible in simulations run with smaller population size, although modularity is maintained more frequently in such small populations (see Additional File 5). The latter observation is in line with theoretical predictions resulting from studies of simple models of gene regulatory pathways [15].

**Figure 5:**
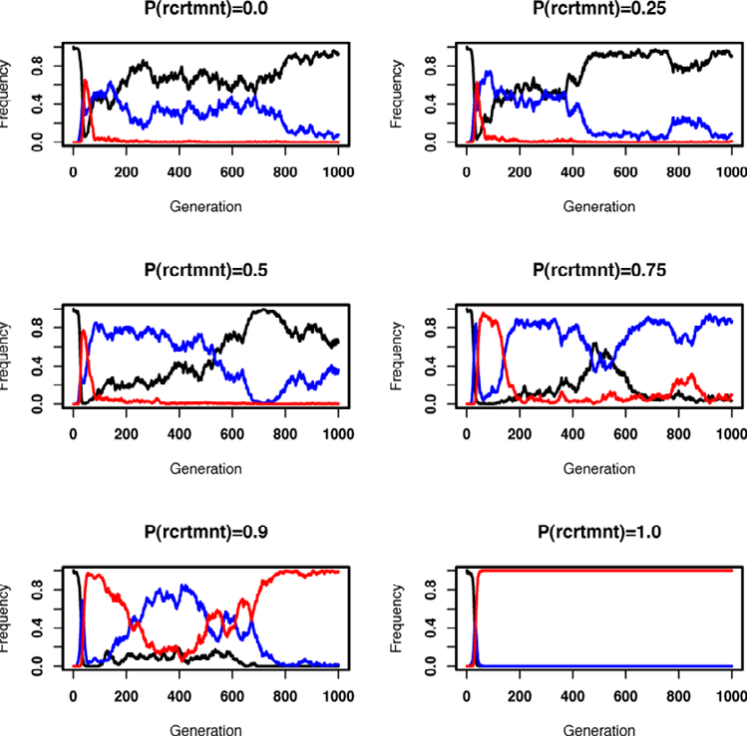
Frequency of different pathway structures during the course of evolution. Different panels show results from sample simulations with increasing probability for protein recruitment (*P(rcrtmnt*)) in expense of interaction formation (i.e. results from one of the runs used to create Figure 4). Red, blue and black lines show the frequency of modular, crosstalk, and complex pathways (see the legend of Figure 4 for pathway types). Note, that measurements are taken after mutations but before selection, hence there is a small fraction of unconnected pathways at each generation (not shown on the graph).

Analysis of the distribution of pathway types over the entire evolutionary simulation, we get a clearer picture of the relation between mutational events and modularity. As shown in Figure 5, modular pathways emerge relatively quickly in the population regardless of the relative rate of protein recruitment and interaction formation. However, in presence of the latter process modular pathways are quickly replaced by pathways with crosstalk or complex pathways. Note that while the distribution of modular pathways change in the population, the average fitness remains high (see Additional File 3). Analyzing the effects of different mutational processes on pathway structure, we find that transitions from modular pathways to pathways with crosstalk are extensively caused by interaction addition (data not shown). The reverse transitions, resulting in modular pathways, are solely driven by protein and interaction loss. Hence, the emergence and maintenance of functional modules is mostly determined by the relevant rate of these different mutational processes.

**Figure 6:**
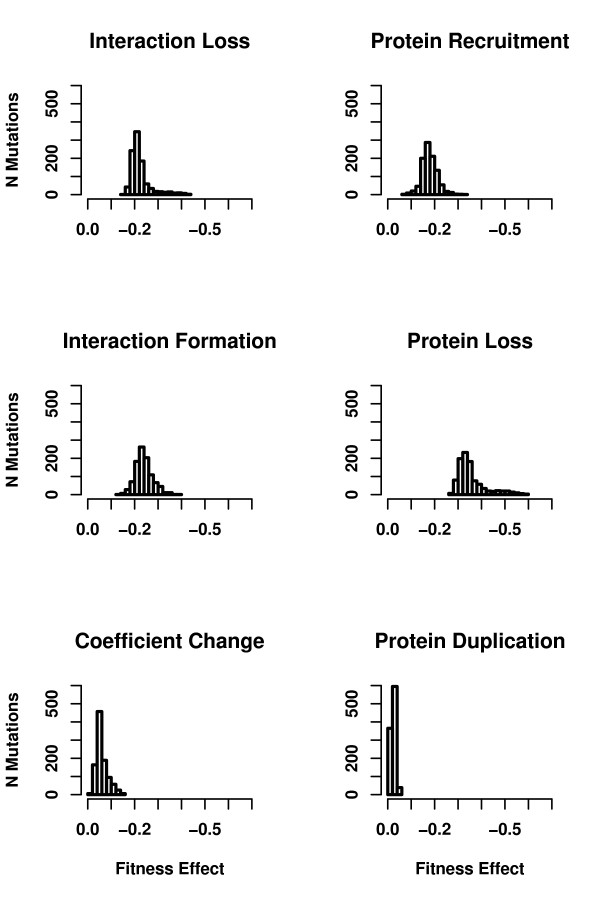
Distribution of fitness effects for each mutation type. Fitness effects of each mutation type are averaged over the entire population. Data is collected and averaged over seven different runs of a simulation where the ratio of protein recruitment probability over the sum of interaction formation and protein recruitment probabilities was 0.5 (one of the simulations used to create Figure 4). Each panel shows the distribution for a different mutational mechanism indicated on the top of the panel.

**Figure 7:**
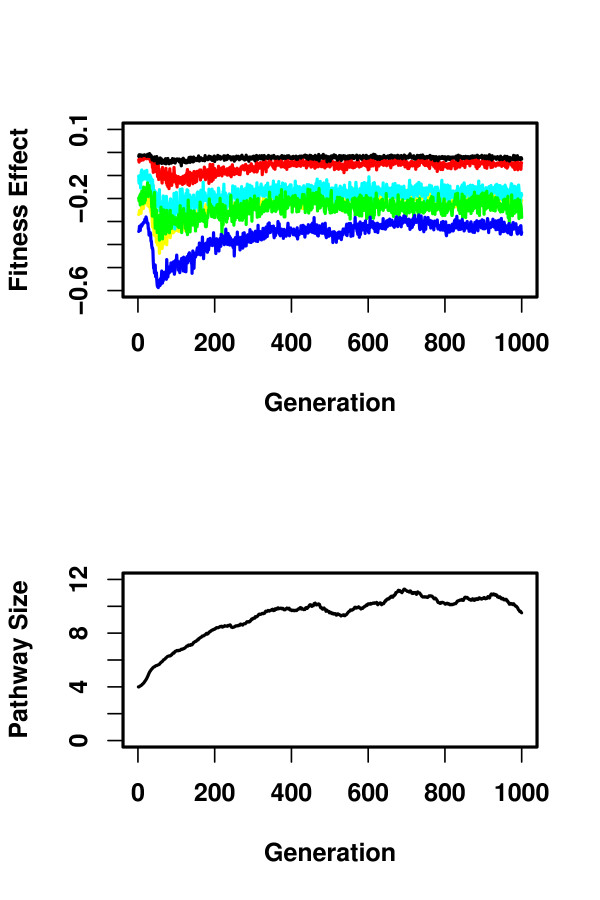
Time course of fitness effects of different mutation types and pathway size. Fitness effects of each mutation type are averaged over the entire population. Data is collected and averaged over seven different runs of a simulation where the ratio of protein recruitment probability over the sum of interaction formation and protein recruitment probabilities was 0.5 (same data as in Figure 6). Different colors indicate different mutation types. Using the notation of Figure 6 we have; black for "Protein Duplication", red for "Coefficient Change", blue for "Protein Loss", green for "Interaction Formation", cyan for "Protein Recruitment", and yellow for "Interaction Loss".

Another key mutational process is duplication of proteins already participating in the pathway. Without duplication, there is no possibility of functional modules emerging. For example, new receptors can only be created through duplication in the model (see *Methods*). Furthermore, duplications push pathways to grow in size and make it possible for the pathway structure to be rearranged towards modularity via other mutational events. Pathway growth (see Additional File 3) occurs despite the higher frequency of protein loss mutations because duplications, and to some extent protein recruitments, are less costly in terms of fitness (see Figure 6). As shown in Figure 7, the average fitness cost of duplications remains low over the entire evolution and does not depend on pathway size. On the other hand, negative fitness effects of other mutational events, especially of mutations leading to protein loss from the pathway, are more pronounced when pathways are smaller. These findings are inline with previous studies analyzing pathway growth in similar models [18]. Similarly, employing a high fitness cost for additional proteins in the model prohibits pathway growth and emergence of modularity (see *Methods*).

To summarize, results from these evolutionary simulations suggest the following scenario for the evolution of functional modules. Simple, non-specific pathways that arose early in evolution would grow in size due to low fitness costs associated with protein duplication and recruitment events. As pathways grow, mutations leading to loss of proteins or their interaction would lead to rearrangement of the pathway structure resulting in the emergence of functional modules. This process happens surprisingly easily and does not require a complex selective pressure. In fact, we find that functional modules emerge even with an alternative fitness function that is simply based on the ability of the pathway to respond to two signals (i.e. no additional reward for response separation). This supports a neutralistic view for the emergence of modularity, as envisioned in regulatory pathways [15]. Once emerged, functional modules would then be maintained depending on the frequency of mutations leading to formation of new interactions among proteins participating in the pathway. This process causes modular pathways to drift towards complex pathways and crosstalk, which provide equally fit solutions as their modular neighbors.

The results leading to this scenario are obtained under the assumption that evolution starts with a specific ancestral pathway structure. To analyze how this initial pathway structure affects the emergence of modularity, we run additional simulations with an initial heterogeneous population composed of 1000 random pathways that contain two, three or five intermediary proteins. As before, we assume that interaction formation mutations between proteins already participating in the pathway can be neglected (i.e. we model all interaction forming mutations as protein recruitment). We find that the exact structure of the ancestral pathway does not have a large effect on the emergence of modularity as long as the pathway is small (see Additional File 4). For larger pathways, it becomes more difficult for mutational events to restructure them towards modularity as the topology space available to a pathway increases exponentially with pathway size. As seen from the high variance in the results of these simulations, the outcome of the evolution for such larger pathways depends highly on the location of the initial pathway in the topology space and the distribution of functional pathways (i.e. pathways that are able to produce separated responses) in this space. This indicates an important role for such neighbor relations in the evolution of system level properties and is inline with previous theoretical studies [19].

## Conclusion

This study provides a simple and biologically plausible explanation for the emergence of modularity in biological signaling pathways. According to this explanation, functional modules specializing in processing one of the multiple signals an organism could encounter emerges readily under a constant selective pressure. The driving processes behind such emergence are protein duplication and recruitment events leading to pathway growth, and loss of proteins and their interactions leading to rearrangement of pathway topology. Once arisen, the probability that such functional modules will be maintained will depend on the frequency of mutations leading to formation of interactions among proteins already participating in the pathway and the fitness of resulting pathways. In other words, the extent of modularity in a specific pathway will mainly depend on the relevant rates of different mutational mechanisms and how functional pathway topologies are distributed over the entire topology space for a given function.

These findings are highly relevant for our understanding of modularity in biological systems, and for applying such understanding to mimic biology in engineering applications (such as in [13]). Firstly, they validate the previous arguments that modularity can emerge readily in biological pathways [14,15] without any need for complex selective pressure. Here we focus on simple mutational processes to provide a mechanistic explanation for the emergence of modularity. We note that more complex processes like horizontal gene transfer, that we did not consider here, could facilitate such emergence as it is found that most of such events involve transfer of entire receptor-effector pairs rather than individual proteins in bacteria [20]. Secondly, the results indicate that although modularity can emerge easily, it is difficult to maintain, as nonmodular pathways (e.g. those with crosstalk) can be equally capable of achieving functionality. Examples for both types of pathways are abundant in biology with two-component signaling pathways of bacteria providing a particularly well-studied case. A systematic study of these systems in the model organism *Escherichia coli *show that the core element of these pathways, the histidine kinase – response regulator pair, can be highly specific (assuring an isolated and modular pathway) or not, allowing crosstalk among different pairs (and signaling pathways) [21]. Here, we specifically looked at how the relevant rates of different mutational processes and the distribution of pathway structures in topology space affect this balance between maintenance of modularity and emergence of crosstalk. An equally important role could be played by the secondary fitness benefits of modularity, such as increases in evolvability [22] or robustness.

The presented scenario for the evolution of signaling pathways is in its essence similar to the one put forward for the evolution of metabolic pathways. According to that theory, current day metabolic pathways with specialized enzymes have evolved, from an ancestral pathway containing non-specific ones, under constant selective pressure for high metabolic yield [23]. Similarly, we find that distinct signaling pathways (i.e. functional modules), specific for processing a single signal, can emerge from an ancestral system containing non-specific receptors, phosphotases and kinases. The resulting modular pathways are underlined by high specificity among components and little or no crosstalk. Such specificity is achieved by different mechanisms in nature including kinetic preference [21,24,25], scaffolding [26], and spatial localization [27] and allows biological systems to ensure signaling fidelity. We believe that combining the knowledge on such molecular-level mechanisms with evolutionary studies of system level properties [15,18,19,28-34] will be crucial for achieving a complete system level understanding in biology.

## Methods

### Pathway model

Here, we use a generic mathematical model of biological pathways, which captures their basic properties. The model has been explained in detail previously [18,35,36]. In brief, it considers a pathway as a collection of interacting proteins, each of which can exist either in an active (P_i_*) or inactive (P_i_) state. Initially, proteins exist in equilibrium between these two states. Proteins that are in the active state can interact with another protein and influence its equilibrium. There are many different biochemical mechanisms that make such influence possible including phosphorylation, methylation, and physical contact. This model does not distinguish among these different mechanisms and uses a simple interaction coefficient to describe activation and deactivation of proteins by other proteins. Thus, for a given protein *i *the chemical equilibrium between active and inactive states is defined as:

Pi⇄∑jlij[pj∗]∑jkij[pj∗]Pi∗
 MathType@MTEF@5@5@+=feaafiart1ev1aaatCvAUfKttLearuWrP9MDH5MBPbIqV92AaeXatLxBI9gBaebbnrfifHhDYfgasaacPC6xNi=xI8qiVKYPFjYdHaVhbbf9v8qqaqFr0xc9vqFj0dXdbba91qpepeI8k8fiI+fsY=rqGqVepae9pg0db9vqaiVgFr0xfr=xfr=xc9adbaqaaeGacaGaaiaabeqaaeqabiWaaaGcbaGaemiuaa1aaSbaaSqaaiabdMgaPbqabaGcdaGdnaWcbaWaaabuaeaacqWGRbWAdaWgaaadbaGaemyAaKMaemOAaOgabeaaliabcUfaBjabdchaWnaaDaaameaacqWGQbGAaeaacqGHxiIkaaWccqGGDbqxaWqaaiabdQgaQbqab4GaeyyeIuoaaSqaamaaqafabaGaemiBaW2aaSbaaWqaaiabdMgaPjabdQgaQbqabaWccqGGBbWwcqWGWbaCdaqhaaadbaGaemOAaOgabaGaey4fIOcaaSGaeiyxa0fameaacqWGQbGAaeqaoiabggHiLdaakiaawkzicaGLqgcacqWGqbaudaqhaaWcbaGaemyAaKgabaGaey4fIOcaaaaa@51CF@

where [Pj∗
 MathType@MTEF@5@5@+=feaafiart1ev1aaatCvAUfKttLearuWrP9MDH5MBPbIqV92AaeXatLxBI9gBaebbnrfifHhDYfgasaacPC6xNi=xH8viVGI8Gi=hEeeu0xXdbba9frFj0xb9qqpG0dXdb9aspeI8k8fiI+fsY=rqGqVepae9pg0db9vqaiVgFr0xfr=xfr=xc9adbaqaaeGacaGaaiaabeqaaeqabiWaaaGcbaGaemiuaa1aa0baaSqaaiabdQgaQbqaaiabgEHiQaaaaaa@2F75@] represents the concentration of active form of protein *j *and *k*_*ij *_and *l*_*ij *_represent the strength of the interaction between protein *i *and *j*. It is assumed that in case there is an energy requirement for interaction of protein *i *with *j*, it is provided from outside sources such as high-energy molecules. The influence of each protein on other proteins can only be activating or deactivating (i.e. *k*_*ij*_·*l*_*ij *_= 0) and proteins do not influence their own equilibrium (i.e. *k*_*ii *_= *l*_*ii *_= 0). In other words, processes such as autophosphorylation or intrinsic phosphotase activity are not considered in this model.

In addition to the general reaction scheme shown in (1), some proteins in the pathway can interact with signaling molecules (i.e. ligand), hence acting as receptors. Further, there are proteins – so called effectors – that are assumed to mediate the physiological response of the pathway in their active state. In a natural pathway this response can have various forms depending on the effector and can range from transcriptional control to enzymatic activity. It is assumed that effectors act solely as response regulators and do not influence the equilibrium state of other proteins in the pathway (i.e. they can not act on other proteins). To summarize, this model defines a biological pathway by a given number of proteins and a set of coefficients defining their interactions. Pathway response to one or more changing ligand concentrations can thus be obtained by solving the set of differential equations resulting from the collection of reactions as shown in (1):

d[Pi]dt=[[Pi∗]⋅∑jlij⋅[Pj∗]]−[[Pi]⋅(ais⋅[Ls]+∑jkij⋅[Pj∗])]
 MathType@MTEF@5@5@+=feaafiart1ev1aaatCvAUfKttLearuWrP9MDH5MBPbIqV92AaeXatLxBI9gBaebbnrfifHhDYfgasaacPC6xNi=xI8qiVKYPFjYdHaVhbbf9v8qqaqFr0xc9vqFj0dXdbba91qpepeI8k8fiI+fsY=rqGqVepae9pg0db9vqaiVgFr0xfr=xfr=xc9adbaqaaeGacaGaaiaabeqaaeqabiWaaaGcbaWaaSaaaeaacqWGKbazcqGGBbWwcqWGqbaudaWgaaWcbaGaemyAaKgabeaakiabc2faDbqaaiabdsgaKjabdsha0baacqGH9aqpdaWadaqaaiabcUfaBjabdcfaqnaaDaaaleaacqWGPbqAaeaacqGHxiIkaaGccqGGDbqxcqGHflY1daaeqbqaaiabdYgaSnaaBaaaleaacqWGPbqAcqWGQbGAaeqaaOGaeyyXICTaei4waSLaemiuaa1aa0baaSqaaiabdQgaQbqaaiabgEHiQaaakiabc2faDbWcbaGaemOAaOgabeqdcqGHris5aaGccaGLBbGaayzxaaGaeyOeI0YaamWaaeaacqGGBbWwcqWGqbaudaWgaaWcbaGaemyAaKgabeaakiabc2faDjabgwSixpaabmaabaGaemyyae2aaSbaaSqaaiabdMgaPjabdohaZbqabaGccqGHflY1cqGGBbWwcqWGmbatdaWgaaWcbaGaem4Camhabeaakiabc2faDjabgUcaRmaaqafabaGaem4AaS2aaSbaaSqaaiabdMgaPjabdQgaQbqabaGccqGHflY1cqGGBbWwcqWGqbaudaqhaaWcbaGaemOAaOgabaGaey4fIOcaaOGaeiyxa0faleaacqWGQbGAaeqaniabggHiLdaakiaawIcacaGLPaaaaiaawUfacaGLDbaaaaa@7A2F@

where [*L*_*s*_] and *a*_*is *_stand for the concentration of ligand *s *and its effect on protein *i *respectively. Note, that the total concentration of each protein [Pitot
 MathType@MTEF@5@5@+=feaafiart1ev1aaatCvAUfKttLearuWrP9MDH5MBPbIqV92AaeXatLxBI9gBaebbnrfifHhDYfgasaacPC6xNi=xH8viVGI8Gi=hEeeu0xXdbba9frFj0xb9qqpG0dXdb9aspeI8k8fiI+fsY=rqGqVepae9pg0db9vqaiVgFr0xfr=xfr=xc9adbaqaaeGacaGaaiaabeqaaeqabiWaaaGcbaGaemiuaa1aa0baaSqaaiabdMgaPbqaaiabdsha0jabd+gaVjabdsha0baaaaa@32CD@] is constant and set to one (i.e. [Pi∗
 MathType@MTEF@5@5@+=feaafiart1ev1aaatCvAUfKttLearuWrP9MDH5MBPbIqV92AaeXatLxBI9gBaebbnrfifHhDYfgasaacPC6xNi=xH8viVGI8Gi=hEeeu0xXdbba9frFj0xb9qqpG0dXdb9aspeI8k8fiI+fsY=rqGqVepae9pg0db9vqaiVgFr0xfr=xfr=xc9adbaqaaeGacaGaaiaabeqaaeqabiWaaaGcbaGaemiuaa1aa0baaSqaaiabdMgaPbqaaiabgEHiQaaaaaa@2F73@] = 1 - [*P*_*i*_]). Also, the maximum value that ligand concentrations and interaction coefficients can attain is set to one for computational ease.

To assess the response of a given pathway, the model is initiated with equal amounts of active and inactive proteins (i.e. [Pi∗
 MathType@MTEF@5@5@+=feaafiart1ev1aaatCvAUfKttLearuWrP9MDH5MBPbIqV92AaeXatLxBI9gBaebbnrfifHhDYfgasaacPC6xNi=xH8viVGI8Gi=hEeeu0xXdbba9frFj0xb9qqpG0dXdb9aspeI8k8fiI+fsY=rqGqVepae9pg0db9vqaiVgFr0xfr=xfr=xc9adbaqaaeGacaGaaiaabeqaaeqabiWaaaGcbaGaemiuaa1aa0baaSqaaiabdMgaPbqaaiabgEHiQaaaaaa@2F73@] = [*P*_*i*_] = 0.5). Then, the system is allowed to equilibrate into a steady state in absence of any signal (i.e. the system of differential equations resulting from [2] is integrated, using the 4^th ^order Runga-Kutta algorithm with step size equal to one, until the point where total change in protein concentrations is below 10^-15 ^and an eigenvalue analysis indicates stability). Once the system is stable, the integration is continued from steady state protein concentrations and two separate signals are introduced (a Gaussian curve with a standard deviation of 10). After introduction of the first signal, the system is integrated until it reaches steady state again, before the second signal is introduced (see Figure 1). The integration is stopped after both signals have been introduced and system reached steady state again (or after a total of 10.000 integration steps have passed). The pathway response is then deduced from the active effector concentrations recorded during integration under ligand presence (shown as a gray area in Figure 1) and is used to calculate the fitness of the pathway as explained below. Systems that do not reach steady state before or after the introduction of signals, are considered unstable and receive a fitness of zero. To avoid any effects of numerical artifacts on the integration process concentrations smaller than 10^-9 ^are set to zero.

### Evolutionary simulations

In order to study the evolution of modularity, a specific ancestral pathway is defined. It contains two effectors, a receptor and an intermediary protein. The latter two are assumed to act as a "global" activator and deactivator respectively. In other words, both proteins are highly non-specific; the receptor is activated by all present signals and relays this activity to the effectors, and the intermediary protein inhibits both the receptor and the effectors (see Figure 1). This pathway could be thought of as the predecessor of bacterial two-component signaling pathways [37], where the receptor and the intermediary protein would correspond to a non-specific histidine kinase and phosphotase respectively.

An initial homogenous population of 1000 ancestral pathways is evolved for 1000 generations (running simulations up to 2000 generations gave qualitatively similar results to those presented in the main text as shown in Additional File 5). During evolution, pathways are subjected to selection for responding separately to the two signals presented at different times of the integration process as shown in Figure 1. Based on this selection criterion, pathway fitness (*F*) is defined as:

F=14⋅[(E1A+E2B)+(E1A+E2B−E1B−E2A)]−n⋅c
 MathType@MTEF@5@5@+=feaafiart1ev1aaatCvAUfKttLearuWrP9MDH5MBPbIqV92AaeXatLxBI9gBaebbnrfifHhDYfgasaacPC6xNi=xI8qiVKYPFjYdHaVhbbf9v8qqaqFr0xc9vqFj0dXdbba91qpepeI8k8fiI+fsY=rqGqVepae9pg0db9vqaiVgFr0xfr=xfr=xc9adbaqaaeGacaGaaiaabeqaaeqabiWaaaGcbaGaemOrayKaeyypa0ZaaSaaaeaacqaIXaqmaeaacqaI0aanaaGaeyyXIC9aamWaaeaacqGGOaakcqWGfbqrdaqhaaWcbaGaeGymaedabaGaemyqaeeaaOGaey4kaSIaemyrau0aa0baaSqaaiabikdaYaqaaiabdkeacbaakiabcMcaPiabgUcaRiabcIcaOiabdweafnaaDaaaleaacqaIXaqmaeaacqWGbbqqaaGccqGHRaWkcqWGfbqrdaqhaaWcbaGaeGOmaidabaGaemOqaieaaOGaeyOeI0Iaemyrau0aa0baaSqaaiabigdaXaqaaiabdkeacbaakiabgkHiTiabdweafnaaDaaaleaacqaIYaGmaeaacqWGbbqqaaGccqGGPaqkaiaawUfacaGLDbaacqGHsislcqWGUbGBcqGHflY1cqWGJbWyaaa@55E7@

where E1A
 MathType@MTEF@5@5@+=feaafiart1ev1aaatCvAUfKttLearuWrP9MDH5MBPbIqV92AaeXatLxBI9gBaebbnrfifHhDYfgasaacPC6xNi=xH8viVGI8Gi=hEeeu0xXdbba9frFj0xb9qqpG0dXdb9aspeI8k8fiI+fsY=rqGqVepae9pg0db9vqaiVgFr0xfr=xfr=xc9adbaqaaeGacaGaaiaabeqaaeqabiWaaaGcbaGaemyrau0aa0baaSqaaiabigdaXaqaaiabdgeabbaaaaa@2F0E@ and E2A
 MathType@MTEF@5@5@+=feaafiart1ev1aaatCvAUfKttLearuWrP9MDH5MBPbIqV92AaeXatLxBI9gBaebbnrfifHhDYfgasaacPC6xNi=xH8viVGI8Gi=hEeeu0xXdbba9frFj0xb9qqpG0dXdb9aspeI8k8fiI+fsY=rqGqVepae9pg0db9vqaiVgFr0xfr=xfr=xc9adbaqaaeGacaGaaiaabeqaaeqabiWaaaGcbaGaemyrau0aa0baaSqaaiabikdaYaqaaiabdgeabbaaaaa@2F10@ (E1B
 MathType@MTEF@5@5@+=feaafiart1ev1aaatCvAUfKttLearuWrP9MDH5MBPbIqV92AaeXatLxBI9gBaebbnrfifHhDYfgasaacPC6xNi=xH8viVGI8Gi=hEeeu0xXdbba9frFj0xb9qqpG0dXdb9aspeI8k8fiI+fsY=rqGqVepae9pg0db9vqaiVgFr0xfr=xfr=xc9adbaqaaeGacaGaaiaabeqaaeqabiWaaaGcbaGaemyrau0aa0baaSqaaiabigdaXaqaaiabdkeacbaaaaa@2F10@ and E2B
 MathType@MTEF@5@5@+=feaafiart1ev1aaatCvAUfKttLearuWrP9MDH5MBPbIqV92AaeXatLxBI9gBaebbnrfifHhDYfgasaacPC6xNi=xH8viVGI8Gi=hEeeu0xXdbba9frFj0xb9qqpG0dXdb9aspeI8k8fiI+fsY=rqGqVepae9pg0db9vqaiVgFr0xfr=xfr=xc9adbaqaaeGacaGaaiaabeqaaeqabiWaaaGcbaGaemyrau0aa0baaSqaaiabikdaYaqaaiabdkeacbaaaaa@2F12@) stand for the maximum of the difference in active effector concentrations between their pre- (i.e. steady state) and post-signal values in the time bracket from introduction of signal A (B) until system reaches steady state again (as described above, also see Figure 1). Further, *n *is the number of proteins in the pathway and *c *is the fitness cost of each protein. The first part of the fitness function rewards pathways ability to respond to the two signals separately through the two effectors. The second part gives smaller pathways a fitness advantage, the extent of which is controlled by the parameter *c *(for the reported results *c *was 0.001). Presented results hold for *c *values as high as 0.1. At such high fitness cost, protein additions are rarely permitted, keeping pathways from growing in size and limiting the chances for emergence of modularity (see *Results and discussion*). Note that division by four is only to scale fitness between zero and one, allowing it to be used directly as replication probability in the evolutionary simulations (see below).

The same fitness function is used throughout the total duration of an evolutionary simulation, representing a constant selective pressure on the pathways as they evolve. The specific function shown in equation 3 is an *ad-hoc *choice that is biologically plausible. There can be many different fitness functions, that are similar to this one and that could lead to modularity. In fact, even simulations with the simplest scheme where fitness equaled pathways ability to respond to the two signals (i.e. *F *= 0.5·(E1A
 MathType@MTEF@5@5@+=feaafiart1ev1aaatCvAUfKttLearuWrP9MDH5MBPbIqV92AaeXatLxBI9gBaebbnrfifHhDYfgasaacPC6xNi=xH8viVGI8Gi=hEeeu0xXdbba9frFj0xb9qqpG0dXdb9aspeI8k8fiI+fsY=rqGqVepae9pg0db9vqaiVgFr0xfr=xfr=xc9adbaqaaeGacaGaaiaabeqaaeqabiWaaaGcbaGaemyrau0aa0baaSqaaiabigdaXaqaaiabdgeabbaaaaa@2F0E@ + E2B
 MathType@MTEF@5@5@+=feaafiart1ev1aaatCvAUfKttLearuWrP9MDH5MBPbIqV92AaeXatLxBI9gBaebbnrfifHhDYfgasaacPC6xNi=xH8viVGI8Gi=hEeeu0xXdbba9frFj0xb9qqpG0dXdb9aspeI8k8fiI+fsY=rqGqVepae9pg0db9vqaiVgFr0xfr=xfr=xc9adbaqaaeGacaGaaiaabeqaaeqabiWaaaGcbaGaemyrau0aa0baaSqaaiabikdaYaqaaiabdkeacbaaaaa@2F12@)) resulted in emergence of modular pathways. This indicates that for the presented analysis, which explores the effects of mechanistic processes on modularity, the exact choice of the fitness function is not crucial.

Throughout the evolutionary simulation, each generation is created from the previous one by randomly selecting individuals for replication with replacement. Randomly selected pathways replicate with a probability proportional to their fitness, and undergo mutations per protein with a certain probability (for the reported results this probability was 0.05, and simulations with 0.1 and 0.005 produce qualitatively equivalent results). Such mutations can cause one of the following with the given probabilities: loss of an existing interaction (*P *= 0.4) or protein (*P *= 0.2) in the pathway, formation of an interaction (*P *= 0.1), duplication of an existing protein (*P *= 0.1) or variation in the coefficient of a randomly selected interaction (*P *= 0.2). These probabilities represent the commonly accepted view that deleterious mutations are more frequent.

All these evolutionary mechanisms are biologically plausible. Except for duplications, we consider all these mechanisms resulting from one or multiple point mutations affecting protein structure and function. For interaction-loss and – adjustment events we assume mutations lead to changes in the binding surface of one protein leading to loss of an interaction or changes in its efficiency. Simulating these events involved randomly selecting two interacting proteins from the pathway, and adjusting the associated coefficient describing their interaction (i.e. the coefficient is set to zero or adjusted by a random percentage). We consider on which protein the simulated mutation has occurred and account for its effects on duplicates of the selected proteins. For example, if protein *i *lose its ability to interact with protein *j *because of mutations happened on itself (on protein *j*), then it (protein *j*) will also lose its ability to interact with duplicates of protein *j *(protein *i*). Duplication events are assumed to result from larger genomic mutations, and are simulated by adding a new protein to the pathway with exactly those interactions as a randomly selected protein from the pathway (the duplicated protein). A duplicate protein is treated as such until it receives another mutation, after which, it is treated as a unique protein.

Mutations resulting in formation of an interaction require special treatment as such an interaction can arise among proteins already participating in a given pathway (i.e. interaction formation), or between a non-participating and participating one (i.e. protein recruitment). To account for these different routes, and to evaluate their effects on the evolution of modularity, we run additional simulations where single interaction additions are simulated as protein recruitment or formation of interaction among existing proteins with a certain probability. As discussed in *Results and discussion *section the ratio between the relevant rates of these two routes affect modularity but not fitness and pathway size (see Additional File 3). Simulating mutations leading to interaction formation involved randomly selecting two proteins. Both or only one of these proteins are selected from the pathway in case of interaction formation and protein recruitment respectively. For protein recruitment we consider the second protein to be one of the many existing proteins in the cell that are not participating in the pathway until that point. In case of both proteins being selected from the pathway (interaction formation), the selection procedure also included signals. Furthermore, the selection procedure did not allow selection of non-receptor proteins and a signal or selection of two participating proteins that are duplicates of the same protein or of each other (as this would correspond to the formation of a self-interaction). As such, the selection processes allows for the possibility for a receptor to start interacting with a signal that it did not interact with before, but does not allow non-receptor proteins to turn into receptors. In other words, the only way for new receptors to arise in this model is through duplication of existing ones. Once two proteins are selected, an interaction is created among them by randomly selecting a coefficient from the interval [-1,1]. If a selected interaction coefficient for proteins *i *and *j *was negative (positive) then *l*_*ij *_(*k*_*ij*_) is set to the absolute value of this number and *k*_*ij *_(*l*_*ij*_) is set to zero.

To test for the effects of the initial population composition, we have run additional simulations starting with a randomly generated heterogeneous population. In different runs we initiated the population with pathways composed of two receptors, two effectors, and two, three, or five, intermediate proteins that are randomly connected. The distribution of different pathway types in three sample runs for each condition is shown in Additional File 4. To test for the effects of population size, we have run additional simulations with population size 100 and using different probability for protein recruitment. Results from these runs are shown in Additional File 5 and discussed in the main text.

Throughout the evolutionary simulations pathway fitness, and various pathway properties are recorded. Each simulation is run multiple times to ascertain that the qualitative conclusions made here are robust to stochastic fluctuations inherent in these evolutionary simulations. All simulations are written in C++ and the source code is available from the authors upon request. Sample pathway structures are drawn using Graphviz.

## Authors' contributions

OSS conceived and designed the study, performed the experiments, analyzed the data, contributed reagents/materials/tools, and wrote the paper.

## Supplementary Material

Additional file 1 Fitness plots from additional evolutionary simulations. Plot showing average fitness of the population during six additional evolutionary simulations (indicated with different colors). Each simulation starts with a homogenous population containing only the ancestral pathway and using the same parameters as for the simulation shown in Figure 2. Click here for file

Additional file 2 Sample evolved pathway structures. Cartoon representations of sample pathways, that resulted from an evolutionary simulation where the ratio of protein recruitment probability over the sum of interaction formation and protein recruitment probabilities was 0.5. These samples are chosen to represent different structural pathway types, from top to bottom; modular, crosstalk, and complex (see legend of Figure 4 for pathway types). All shown pathways achieve a fitness level above 0.9 and are able to produce separate signal-response dynamics. Click here for file

Additional file 5 Frequency of different pathway structures in final populations. Plot showing the frequency of different pathway structures in the final generation of the evolutionary simulations with increasing ratio of protein recruitment over the sum of interaction formation and protein recruitment probabilities. The two panels show results from two different simulation conditions; (top) Population Size = 100, N Generations = 1000 (bottom) Population Size = 1000, N Generations = 2000. For each probability ratio, the frequencies are obtained as an average over seven and three different runs for small and large populations respectively. We distinguish among three different structural types for pathways. Pathways where there is a path from each signal to only one effector and the other (modular, solid circles), pathways where there is a path from one of the signals to both effectors (crosstalk, open circles), pathways where there is a path from each signal to each effector (complex, diamonds). Click here for file

Additional file 3 Average fitness and pathway size for different evolutionary simulations. Plot showing the average fitness and pathway size for evolutionary simulations with different ratio of protein recruitment probability over the sum of interaction formation and protein recruitment probabilities (indicated with different colors). Shown values are averaged over seven runs for each simulation. Click here for file

Additional file 4 Frequency of different pathway structures during the course of evolution. Plots showing the frequency of different pathway structures during the course of evolution for three sample simulations starting with an initial random population and using *P(rcrtmnt*) = 1.0 (as in last panel of Figure 5). Rows from top to bottom show results with initial populations composed of random pathways containing six, seven, and nine proteins respectively (see *Methods*). Red, blue and black lines show the frequency of modular, crosstalk, and complex pathways (see the legend of Figure 4 in the main text for pathway types). Click here for file
