# Supplementary material for: Emergence and maintenance of functional modules in signaling pathways
Source: BMC Evol Biol. 2007 Oct 31;7:205. doi: 10.1186/1471-2148-7-205 (PMC2228312; doi:10.1186/1471-2148-7-205)
Supplement: Additional file 1 — Fitness plots from additional evolutionary simulations. Plot showing average fitness of the population during six additional evolutionary simulations (indicated with different colors). Each simulation starts with a homogenous population containing only the ancestral pathway and using the same parameters as for the simulation shown in Figure 2. [file 1471-2148-7-205-S1.doc]

**Additional file 1:**


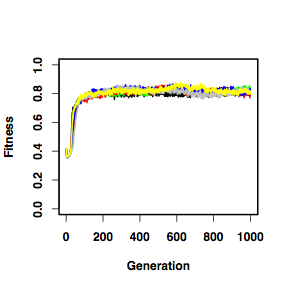


Fitness for six additional runs (indicated with different colors) of an evolutionary simulation starting with a homogenous population containing only the ancestral pathway and using the same parameters as for the simulation shown in Figure 2. Only the average fitness of the population is shown.
