# Supplementary material for: Emergence and maintenance of functional modules in signaling pathways
Source: BMC Evol Biol. 2007 Oct 31;7:205. doi: 10.1186/1471-2148-7-205 (PMC2228312; doi:10.1186/1471-2148-7-205)
Supplement: Additional file 2 — Sample evolved pathway structures. Cartoon representations of sample pathways, that resulted from an evolutionary simulation where the ratio of protein recruitment probability over the sum of interaction formation and protein recruitment probabilities was 0.5. These samples are chosen to represent different structural pathway types, from top to bottom; modular, crosstalk, and complex (see legend of Figure 4 for pathway types). All shown pathways achieve a fitness level above 0.9 and are able to produce separate signal-response dynamics. [file 1471-2148-7-205-S2.doc]

**Additional file 2:**


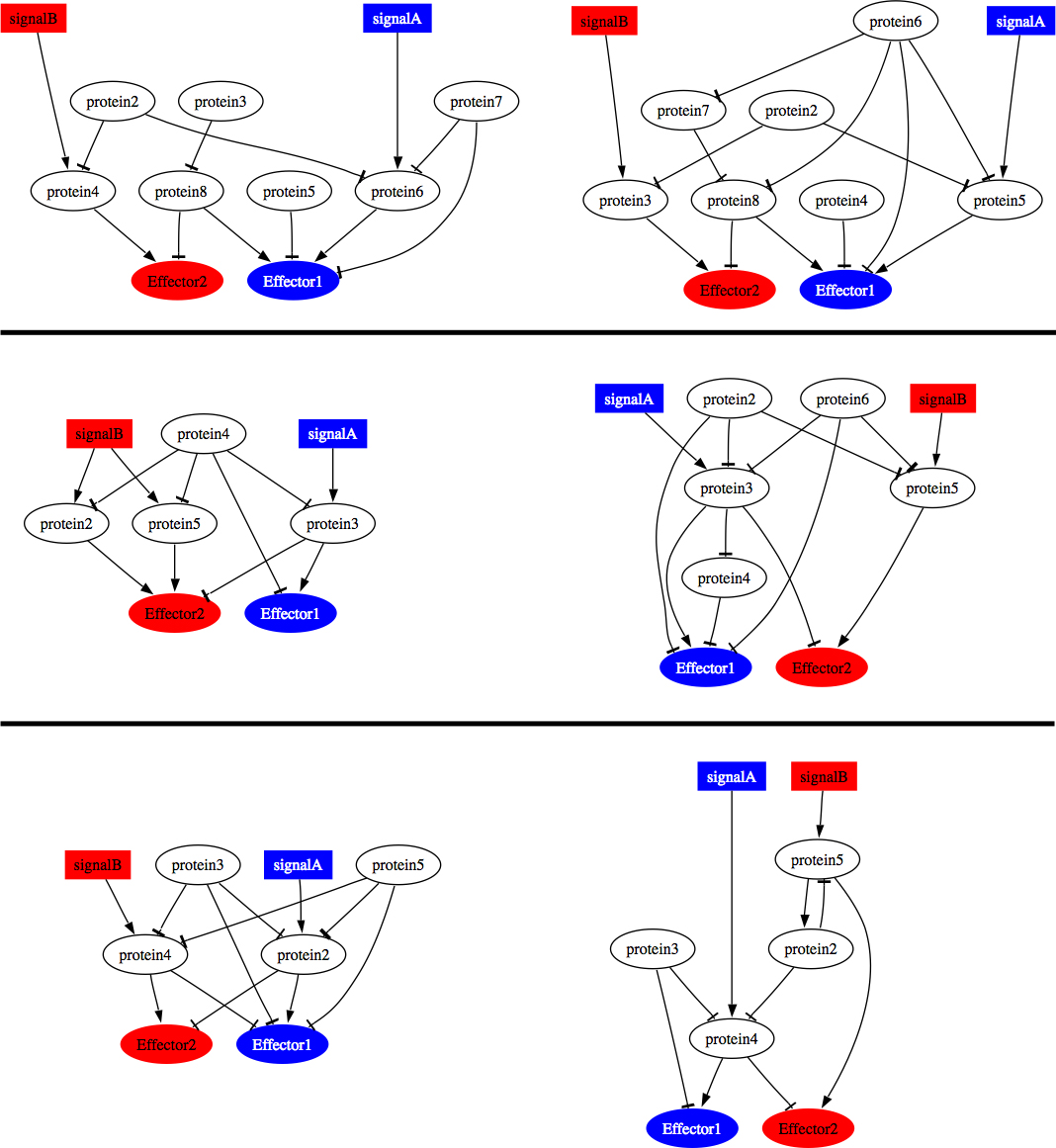


Sample pathways resulting from an evolutionary simulation where the ratio of protein recruitment probability over the sum of interaction formation and protein recruitment probabilities was 0.5. Samples are chosen to represent different structural pathway types, from top to bottom; modular, crosstalk, and complex (see legend of Figure 4 for pathway types). All shown pathways achieve a fitness level above 0.9 and are able to produce separate signal-response dynamics.
