# Supplementary material for: Emergence and maintenance of functional modules in signaling pathways
Source: BMC Evol Biol. 2007 Oct 31;7:205. doi: 10.1186/1471-2148-7-205 (PMC2228312; doi:10.1186/1471-2148-7-205)
Supplement: Additional file 5 — Frequency of different pathway structures in final populations. Plot showing the frequency of different pathway structures in the final generation of the evolutionary simulations with increasing ratio of protein recruitment over the sum of interaction formation and protein recruitment probabilities. The two panels show results from two different simulation conditions; (top) Population Size = 100, N Generations = 1000 (bottom) Population Size = 1000, N Generations = 2000. For each probability ratio, the frequencies are obtained as an average over seven and three different runs for small and large populations respectively. We distinguish among three different structural types for pathways. Pathways where there is a path from each signal to only one effector and the other (modular, solid circles), pathways where there is a path from one of the signals to both effectors (crosstalk, open circles), pathways where there is a path from each signal to each effector (complex, diamonds). [file 1471-2148-7-205-S5.doc]

**Additional file 5:**


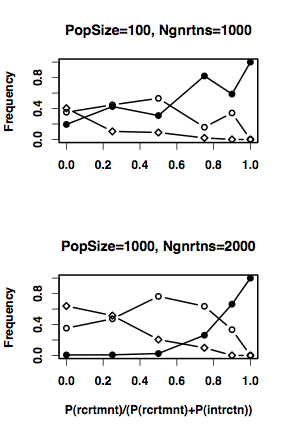


Frequency of different pathway structures in the final generation of the evolutionary simulations with increasing ratio of protein recruitment over the sum of interaction formation and protein recruitment probabilities. The two panels show results from two different simulation conditions; (top) Population Size = 100, N Generations = 1000 (bottom) Population Size = 1000, N Generations = 2000. For each probability ratio, the frequencies are obtained as an average over seven and three different runs for small and large populations respectively. We distinguish among three different structural types for pathways. Pathways where there is a path from each signal to only one effector and the other (modular, solid circles), pathways where there is a path from one of the signals to both effectors (crosstalk, open circles), pathways where there is a path from each signal to each effector (complex, diamonds).
