# Supplementary material for: Emergence and maintenance of functional modules in signaling pathways
Source: BMC Evol Biol. 2007 Oct 31;7:205. doi: 10.1186/1471-2148-7-205 (PMC2228312; doi:10.1186/1471-2148-7-205)
Supplement: Additional file 3 — Average fitness and pathway size for different evolutionary simulations. Plot showing the average fitness and pathway size for evolutionary simulations with different ratio of protein recruitment probability over the sum of interaction formation and protein recruitment probabilities (indicated with different colors). Shown values are averaged over seven runs for each simulation. [file 1471-2148-7-205-S3.doc]

**Additional file 3:**


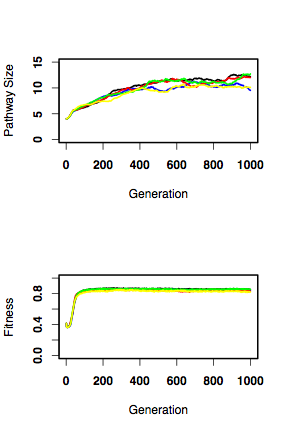


Average fitness and pathway size for evolutionary simulations with different ratio of protein recruitment probability over the sum of interaction formation and protein recruitment probabilities (indicated with different colors). Values are averaged over seven runs for each simulation.
