# Supplementary material for: Emergence and maintenance of functional modules in signaling pathways
Source: BMC Evol Biol. 2007 Oct 31;7:205. doi: 10.1186/1471-2148-7-205 (PMC2228312; doi:10.1186/1471-2148-7-205)
Supplement: Additional file 4 — Frequency of different pathway structures during the course of evolution. Plots showing the frequency of different pathway structures during the course of evolution for three sample simulations starting with an initial random population and using P(rcrtmnt) = 1.0 (as in last panel of Figure 5). Rows from top to bottom show results with initial populations composed of random pathways containing six, seven, and nine proteins respectively (see Methods). Red, blue and black lines show the frequency of modular, crosstalk, and complex pathways (see the legend of Figure 4 in the main text for pathway types). [file 1471-2148-7-205-S4.doc]

**Additional file 4:**


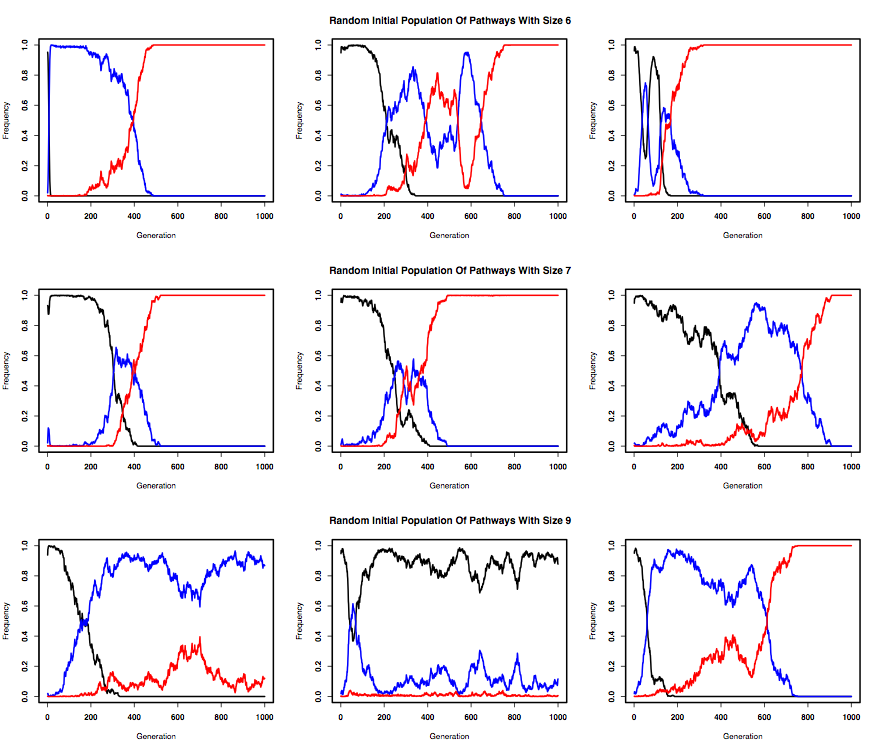


Frequency of different pathway structures during the course of evolution for three sample simulations starting with an initial random population and using *P(rcrtmnt)* = 1.0 (as in last panel of Figure 5). Rows from top to bottom show results with initial populations composed of random pathways containing six, seven, and nine proteins respectively (see *Methods*). Red, blue and black lines show the frequency of modular, crosstalk, and complex pathways (see the legend of Figure 4 in the main text for pathway types).
